# Association Between Follicle-Stimulating Hormone Receptor (FSHR) rs6166 and Estrogen Receptor 1 (ESR1) rs2234693 Polymorphisms and Polycystic Ovary Syndrome Risk, Phenotype, and Reproductive Outcomes in an Infertile Portuguese Population

**DOI:** 10.7759/cureus.35690

**Published:** 2023-03-02

**Authors:** Inês H Vieira, Alexandra F Carvalho, Sandra Almeida Reis, Ana L Carreira, Conceição Dias, Silvana Fernandes, Ana Filipa Ferreira, Dircea Rodrigues, Ana Paula Sousa, João Ramalho-Santos, Ana Cristina Ramalhinho, Mariana Moura Ramos, Isabel Paiva, Paulo Cortesão, Ana Teresa Almeida-Santos

**Affiliations:** 1 Department of Endocrinology, Diabetes, and Metabolism, Coimbra Hospital and University Center, Coimbra, PRT; 2 Reproductive Medicine Unit, Department of Gynecology, Obstetrics, Reproduction, and Neonatology, Coimbra Hospital and University Center, Coimbra, PRT; 3 Center for Neuroscience and Cell Biology (CNC) CIBB, University of Coimbra, Coimbra, PRT; 4 Health Sciences Research Centre (CICS-UBI), University of Beira Interior, Coimbra, PRT; 5 IIIUC - Institute for Interdisciplinary Research, University of Coimbra, Coimbra, PRT; 6 Center for Neuroscience and Cell Biology (CNC), Center for Innovation in Biomedicine and Biotechnology, University of Coimbra, Coimbra, PRT; 7 Faculty of Medicine, University of Coimbra, Coimbra, PRT; 8 Department of Life Sciences, University of Coimbra, Coimbra, PRT; 9 Health Sciences Research Centre (CICS-UBI), University of Beira Interior, Covilhã, PRT; 10 Assisted Reproduction Laboratory, Academic Hospital of Cova da Beira, Covilhã, PRT; 11 Center for Research in Neuropsychology and Cognitive and Behavioral Intervention, University of Coimbra, Coimbra, PRT

**Keywords:** follicle-stimulating hormone, polycystic ovary syndrome, fsh receptor, fsh, estrogen receptor (er), estrogen, anovulation, infertility, single-nucleotide polymorphism, polycystic ovary syndrome (pcos)

## Abstract

Introduction: Polycystic ovary syndrome (PCOS) is a common endocrine disorder often leading to anovulatory infertility. PCOS pathophysiology is still unclear and several potential genetic susceptibility factors have been proposed. The effect of polymorphisms in two genesrelated to follicular recruitment and development, the follicle-stimulating hormone receptor (*FSHR*) and the estrogen receptor 1 (*ESR1*), have been studied in different populations with contradictory results.

Aims: To evaluate the influence of *FSHR *rs6166 (c.2039A>G) and of *ESR1 *rs2234693 (Pvull c.453-397 T > C) polymorphisms on PCOS risk, phenotype, and response to controlled ovarian stimulation (COS).

Materials and methods: Genotyping of the *FSHR *rs6166 and the *ESR1 *rs2234693 polymorphisms was performed in PCOS women and a control group undergoing in vitro fertilization (IVF). Demographic, clinical, and biochemical data, genotype frequency, and IVF outcomes were compared between groups.

Results: We evaluated 88 PCOS women and 80 controls. There was no significant difference in the genotype distribution of *FSHR* rs6166 polymorphism between PCOS women and controls (AA 31.8%/AS 48.9%/SS 19.3% in PCOS women vs AA 37.5%/AS 40.0%/SS 22.5% in controls; p = 0.522). The same was true for the *ESR1* rs2234693 (CC 24.1%/CT 46.0%/TT 29.9% in PCOS women vs CC 18.8%/CT 48.8%/TT 32.5% in controls; p = 0.697). In PCOS women, we found higher follicle-stimulating hormone (FSH) levels on the third day of the menstrual cycle associated with the SS variant of the *FSHR *polymorphism (9.2 vs* *6.2 ± 1.6 and 5.6 ± 1.6 mUI/mL; p = 0.011). We did not find other associations between the baseline hormonal parameters, antral follicle count, and response measures to COS with *FSHR *or *ESR1* genotypes. We found, however, a need for higher cumulative doses of FSH for COS in patients with the SS variant of the *FSHR *rs6166 polymorphism (1860.5 ± 627.8 IU for SSvs* *1498.1 ± 359.3 for AA and 1425.4 ± 474.8 for SA; p = 0.046 and p = 0.046).

Conclusion: Our data suggest that in the population, *FSHR *rs6166and *ESR1 rs2234693* polymorphisms do not influence the risk of developing PCOS nor do they influence the patient’s phenotype and IVF success. However, the SS variant of the *FSHR* rs6166 polymorphism may be associated with FSH resistance requiring higher FSH doses for COS.

## Introduction

Polycystic ovary syndrome (PCOS) is the most common endocrine disorder in women of reproductive age with a prevalence that may be close to 18% [1]. It has adverse reproductive and metabolic implications [2], and it is the most common cause of anovulatory infertility [3]. The pathophysiology of PCOS is still poorly understood; however, several potential genetic susceptibility factors have been identified, including mutations and polymorphisms on the genes involved in the gonadal axis, steroid metabolism, cardiovascular risk, and insulin resistance [4]. In the setting of assisted reproduction techniques (ART), infertile women with PCOS constitute a challenge for controlled ovarian stimulation (COS) [5], and an influence of some polymorphic variations may be at play.

Estrogen and follicle-stimulating hormone (FSH), acting together, lead to an increase in follicle-stimulating hormone receptor (*FSHR*) expression in the granulosa cells contributing to the growth and maturation of ovarian follicles [6].

FSH is important for follicular development and oocyte maturation. Ovarian sensitivity or resistance to exogenous FSH is believed to be influenced by genetic variations related to FSH and its receptor [7]. The *FSHR*, encoded by a gene located in 2q, is a member of the G-protein coupled receptor family [8]. Abnormal *FSHR* function may lead to arrested follicular development, resulting in amenorrhea and high FSH levels [9]. Mutations in the *FSHR *are rare, however, several polymorphisms have been identified [7]. Two variants in exon 10 have received particular attention. In codon 307, a substitution of adenine to guanine in the extracellular domain of the *FSHR* results in a change from threonine (Thr) to alanine (Ala) - *FSHR* rs6165 (c.919G>A, p.Thr307Ala). In codon 680, a substitution of adenine to guanine leads an asparaginase codon to be replaced by a serine codon - *FSHR* rs6166 (c.2039A>G, p.Asn680Ser) [10]. Both variants are in almost complete linkage disequilibrium [11,12]. An association between *FSHR* polymorphism and PCOS risk has been studied with conflicting results [4]. Valkenburg et al. did not find an association between* FSHR* polymorphisms and disease risk but do report an association with its phenotype and hormone levels [12]. There is also some data to support an association between the SS variant of the *FSHR* rs6166 polymorphism with FSH resistance [13] and potentially altered response to ovarian stimulation [11].

Estrogen is essential to trigger the cascade of events that culminates in ovulation as it is a positive regulator of the preovulatory gonadotropin surge [14]. There are data associating estrogen receptor 1 (*ESR1*) gene polymorphisms with ovulatory defects [14] and a possible role in PCOS susceptibility has been suggested [15]. *ESR1*, located on chromosome 6q25.1, has been reported to have two common single nucleotide polymorphisms (SNPs) in the first intron, at restriction enzyme sites: (1) Pvull c.453-397 T > C (rs2234693) (−397 TC, rs2234693, NM_000125.3:c.453-397 T > C) and (2) XbaI (−351 AG, rs9340799, NM_000125.3:c.453-351A > G) [16].

Knowledge of genetic factors that may influence the ovarian response to COS may contribute to the development of pharmacogenetic approaches [17]. In this study, we genotyped the *FSHR *c.2039A>G (rs6166) polymorphism and one of the most common SNP in* ESR1*, *ESR1* Pvull c.453-397 T > C (rs2234693), in a group of women with PCOS and a control group undergoing in vitro fertilization (IVF) treatments. We aimed to investigate if these SNPs influence the risk and phenotype of PCOS in the Portuguese population. We also sought to investigate if either of these polymorphisms was associated with PCOS biochemical phenotype and with the result of COS.

## Materials and methods

Patient selection

We carried out a retrospective observational study of women who underwent infertility treatment in a tertiary fertility clinic between 2013 and 2019.

This study was designed, conducted, and reported following the principles of Good Clinical Practice guidelines and the 1964 Helsinki Declaration and its later amendments. The Hospital Ethics Board granted ethical approval (internal reference number: 171-20), and all participants gave their written informed consent for blood sampling and genetic investigations for these specific targets.

The PCOS group (n = 88) and the control group (n = 80) were constituted of infertile patients undergoing the first cycle of IVF treatment. COS was performed with gonadotropin-releasing hormone (GnRH) antagonist protocol, using recombinant human FSH (rhFSH).

The diagnosis of PCOS was established based on the 2003 European Society of Human Reproduction and Embryology (ESHRE)/American Society for Reproductive Medicine (ASRM) Rotterdam criteria [18]. The presence of PCOS was confirmed by vaginal ultrasound examination.

The control group included age and body mass index (BMI) matched women with regular menstrual cycles. Additionally, the controls were infertile women with tubal obstruction with regular menstrual cycles, no clinical or biochemical signs of hyperandrogenism, and no polycystic ovaries. Patients with male factor infertility, endometrial disease, or severe systemic illness were excluded from both groups.

Genotyping

Genomic deoxyribonucleic acid (DNA) was isolated from peripheral blood collected in ethylenediaminetetraacetic acid-coated tubes on day three of the follicular phase and stored at 4°C until DNA extraction. Extraction of genomic DNA was performed using QIAamp® DNA Blood MiniKit (Qiagen, Hilden, Germany), according to the manufacturer's instructions. QIAamp® DNA Blood Kits provide silica-membrane-based DNA purification from biological fluids (including blood samples). We optimized buffers lyse samples, stabilized nucleic acids, and enhanced selective DNA adsorption to the membrane. Alcohol was added and sample lysates were loaded onto the spin column. Finally, wash buffers were added to remove impurities and purified DNA was then eluted in low-salt buffer. The quantity and purity of each eluted sample were assessed by NanoDrop^TM^ 2000 spectrophotometer (Thermo Fisher Scientific, Waltham, MA).

DNA amplification was carried out using the polymerase chain reaction (PCR) with specific primers to assess *FSHR* rs6166 (c.2039A>G) and *ESR1* rs2234693 (Pvull c.453-397 T > C), as described in Table 1. Specifically, PCR was carried out in a 0.2-mL PCR tube with a 25 μL reaction volume, containing 0.1 μL of template genomic DNA, 1.25 U Taq polymerase (Invitrogen, Thermo Fisher Scientific, Waltham, MA), 0.2 µM deoxynucleotide triphosphates (dNTPs) (Invitrogen), 0.2 µM primer reverse, and 0.2 µM primer forward (Applied Biosystems, Thermo Fisher Scientific, Waltham, MA); the reaction volume was adjusted with nuclease-free water. The amplification reaction for each primer set was conducted in a programmable c1000 Thermal Cycler (Bio-Rad Laboratories, Hercules, CA). Nuclease-free water was used instead of genomic DNA as a blank to check for DNA contamination.

**Table 1:** Summary of the studied single nucleotide polymorphisms PCR: polymerase chain reaction.

Restriction endonucleases enzymes	Primer sequence	Included polymorphism			PCR program
		Product size	DNA variation	Sequence variation	
BseNI	Primer F 5´TTT GTG GTC ATC TGT GGC TGG 3` - Primer R 5`CAA AGG CAA GGA CTG AAT TAT CAT T 3´	520 bp	rs6166	c.2039A>G	94°C, 5 min, 1 cycle; 94°C, 1 min; 60°C 1 min and 72°C 1 min, 40 cycles; 72°C, 1 min, 1 cycle; 12°C, ∞
PvuII	Primer F 5´CGT CCA CCC TAT CTG TAT CTT TTC CTA TTC TCC 3` - Primer R 5`TCT TTC TCT GCC ACC CTG GGG TCG ATT ATC TGA 3´	1373 bp	rs2234693	c.453-397 T > C	95°C, 5 min, 1 cycle; 95°C, 30 sec; 63°C 1 min and 72°C 1 min, 44 cycles; 72°C, 10 min, 1 cycle; 12°C, ∞

Restriction fragment length polymorphism (RFLP) was carried out on the purified PCR products using restriction endonuclease enzymes. To determine the genotype for each sample, PCR products were incubated and digested with the respective restriction enzyme; PvuII (Invitrogen) and BseNI (Thermo Fisher Scientific, Waltham, MA) for *ESR1* rs2234693 (Pvull c.453-397 T > C) and *FSHR* rs6166 (c.2039A>G), respectively, according to the manufacturer’s instructions (Table 2). The samples were then run on a 2% agarose gel containing ethidium bromide (Invitrogen) at 80 V for 75 minutes and visualized by ultraviolet transilluminator (DNA MiniBIS Pro, DNR Bio Imaging System, Neve Yamin, Israel).

**Table 2:** Restriction endonuclease enzymes utilized for RFLP RFLP: restriction fragment length polymorphism; FSHR: follicle-stimulating hormone receptor; ESR1: estrogen receptor 1; bp: base pairs.

Restriction endonucleases enzymes	Hormonal receptor gene		Product size		
		Incubation conditions	Genotype		Band size
BseNi	FSHR	5 min at 65°C; 5 min at 80°C	AA, AS, SS	Homozygotes, heterozygotes, homozygotes	520 bp, 520 + 413 bp, 413 bp
PvuII	ESR1	2 hours at 37°C; 5 min at 80°C	CC, CT, TT	Homozygotes, heterozygotes, homozygotes	1374 bp, 1374+ 936 + 438 bp, 936 + 438 bp

**Figure 1:**
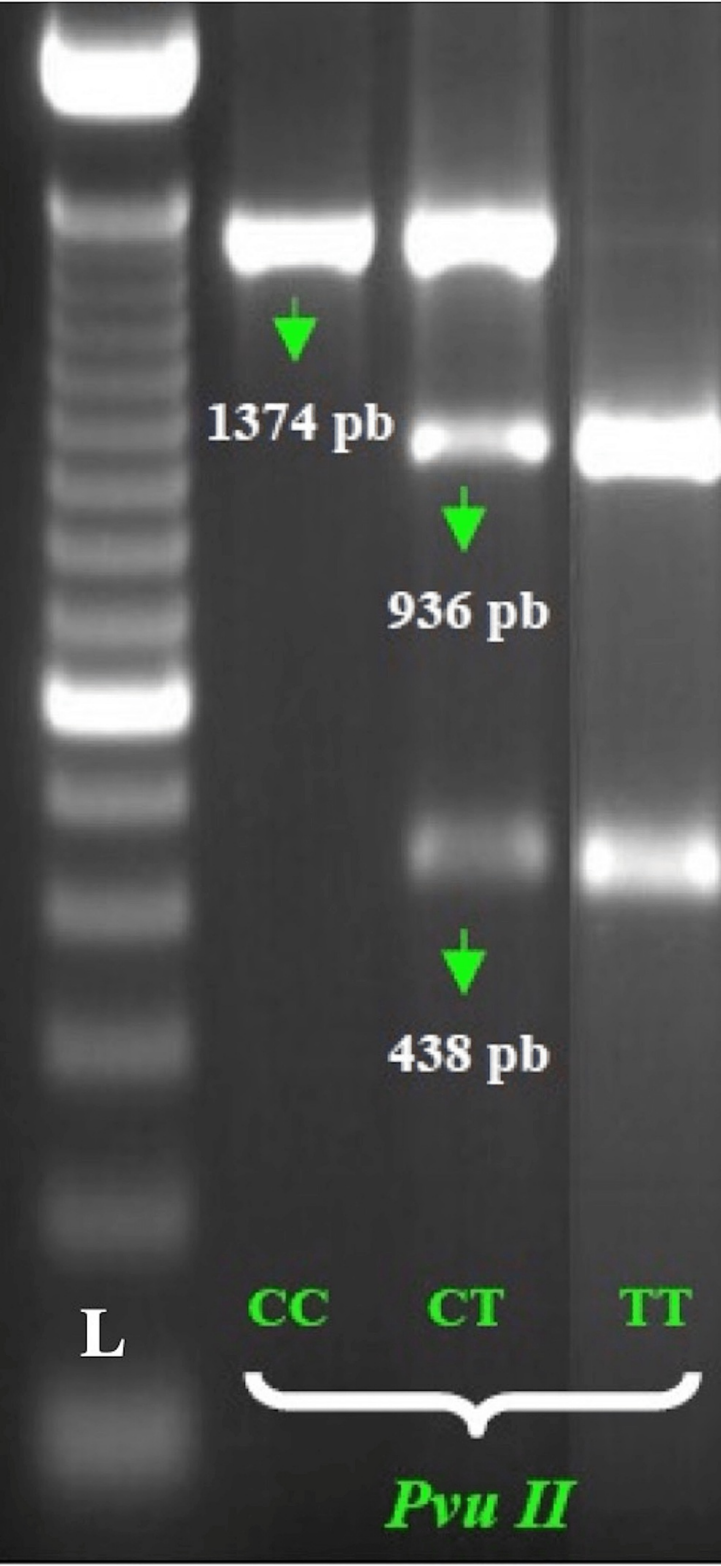
ESR1 rs2234693 genotypes The CC genotype is represented by one band of 1374 bp, the CT genotype is identified by three bands of 1374 bp, 936 bp, and 438 bp, and the TT genotype is represented by two bands of 936 bp and 438 bp. ESR1: estrogen receptor 1.

*ESR1* rs2234693 alters T to C; genotypes are represented as TT and CC for the homozygous type and TC for the heterozygous type. Digestion with PvuII produced a single band of 1374 base pairs (bp) in the CC normal genotype and two bands of 936 and 438 bp in the TT homozygous mutant genotype and three bands of 1374, 936, and 438 bp in the heterozygous CT genotype (Figure 1).

**Figure 2:**
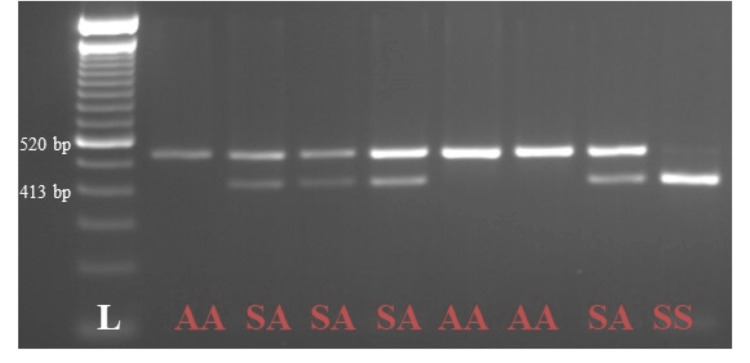
FSHR rs6166 genotypes The AA genotype is represented by one band of 520 bp, the SA genotype is identified by two bands of 520 bp and 413 bp, and the SS genotype is represented by one band of 413 bp. FSHR: follicle-stimulating hormone receptor.

*FSHR* rs6166 leads to a change from A to G. BseNI digestion produces an uncleaved 520 bp fragment that indicates homozygosity for asparagine (Asn/Asn). In contrast, two fragments of 413 and 520 bp indicate heterozygosity (Asn/Ser). The presence of one fragment of 413 bp reveals homozygosity for serine (Ser/Ser), as represented in Figure 2, as previously described in the work of Kuijper and collaborators [13].

Data collection

Clinical information of the patients was collected from the medical records. Collected data included age, BMI, antral follicle count (AFC), plasma concentrations of progesterone, testosterone, anti-müllerian hormone (AMH), FSH, luteinizing hormone (LH), and estradiol in the third and 23rd day of the menstrual cycle, outcomes of the IVF treatment, follicle count in the day of ovulation induction, number and quality of oocytes retrieved, and the number of blastocysts obtained. Information on prior medical history and medication use was also scanned for each of the patients to properly evaluate the above-mentioned inclusion and exclusion criteria.

For the included patients, the results of the analysis of each of the polymorphisms were also inserted in our database.

Data analysis

Statistical analysis was performed using SPSS version 23.0 (IBM Corp., Armonk, NY) and SNPStats. Statistical significance was assumed for p-value < 0.05. Hardy-Weinberg equilibrium was confirmed for both polymorphisms.

For continuous variables, when normal distribution was confirmed (through standardized asymmetry) and homogeneity of variances of variables was noted, Student's t (for two groups) or ANOVA (for ≥ three groups) tests were used to compare groups. In cases where it was not possible to assume a normal distribution, Mann-Whitney U and Kruskal-Wallis tests were used for comparison between two or > two groups, respectively. The chi-square test was performed for categorical variables to test differences in the distribution of two independent variables. Fisher's exact test was used instead whenever expected frequencies below five occurred with enough frequency.

After verifying the required assumptions, linear regression was used whenever there was a need to confirm an independent effect of an independent variable on a continuous dependent variable.

## Results

Sample characterization

Our sample was composed of 168 women, 88 of whom had PCOS. Women from the PCOS and control groups did not differ in age.

A comparison between PCOS and non-PCOS subjects regarding baseline characteristics, baseline hormonal levels, follicle count, and the number of oocyte numbers obtained is summarized in Table 3.

**Table 3:** Clinical and biochemical data of women with PCOS and controls Data expressed as mean ± SD; * p < 0.05 vs control group. PCOS: polycystic ovary syndrome; BMI: body mass index; FSH: follicle stimulating hormone; LH: luteinizing hormone; AMH: anti-müllerian hormone; AFC: antral follicle count; MII: metaphase II oocytes; MI: metaphase I oocytes; GV: germinal vesicle.

	Controls	PCOS	P-value
Age (years)	33.4±11.4	33.1±13.3	0.614
BMI	25.7±5.1	26.8±5.3	0.156
FSH 23^rd^ day (mUI/mL)	3.4±1.5	5.6±2.3	<0.001*
FSH 3^rd^ day (mUI/mL)	7.6±6.4	6.5±4.6	0.001*
LH 23^rd^ day (mUI/mL)	4.2±3.4	12.9±11.1	<0.001*
LH 3^rd^ day (mUI/mL)	4.9±3.2	8.5±6.4	<0.001*
Estradiol 23^rd^ day (pg/mL)	155.2±65.6	109.2±80.4	<0.001*
Estradiol 3^rd^ day (pg/mL)	50.9±27.0	53.6±28.8	0.231
Progesterone (ng/mL)	12.6±7.9	3.8±6.5	<0.001*
Testosterone (ng/mL)	0.4±0.1	0.6±0.5	<0.001*
AMH (ng/mL)	2.8±1.5	7.4±6.1	0.004*
AFC	12.1±4.7	19.1±11.6	<0.001*
Follicle count on the day of ovulation trigger			
10-15 mm	3.3±2.7	6.1±5.0	<0.001*
>15 mm	7.5±3.1	8.4±4.0	0.102
Total	10.8±4.5	14.5±7.3	0.001*
Oocyte number			
MII	6.8±5.1	8.6±6.6	0.086
MI or GV	1.5±1.8	2.2±2.4	0.067
Degenerative or atretic oocyte	1.7±2.8	2.4±3.5	0.032*
Total	10.0±6.7	13.3±9.6	0.023*
Blastocyst number	1.5±2.2	1.5±2.2	0.861

As it was intended by the study design, PCOS and non-PCOS groups were homogeneous in terms of age and body mass index (BMI). However, the groups differed in baseline hormonal levels (FSH and LH on days three and 23 of the menstrual cycle and estradiol, progesterone, and testosterone on day 23). As expected, the AMH, AFC, and follicle count on the day of ovulation induction were higher in the PCOS group. Even though there was a higher number of oocytes obtained in the PCOS group, the oocyte quality was lower. A higher number of degenerative or atretic oocytes was found in the PCOS group.

Evaluation of whether there is an association between *FSHR* and *ESR1* SNPs and PCOS risk

The results of the genotype distribution of *FSHR* rs6166 genotype and *ESR1* rs2234693 are reported in Table 4. We did not identify statistically significant differences in the distribution between PCOS patients and controls.

**Table 4:** Genotype distribution of FSHR rs6166 and ESR1 rs2234693 polymorphisms among PCOS case and control groups PCOS: polycystic ovary syndrome; FSHR: follicle-stimulating hormone receptor; ESR1: estrogen receptor 1.

Genotypes of each polymorphism	Controls	PCOS	P-value
FSHR c.2039A>G (rs6166)			0.522
AA	37.5% (30)	31.8% (28)	(vs AS/SS) 0.516
AS	40.0% (32)	48.9% (43)	(vs AA/SS) 0.279
SS	22.5% (18)	19.3% (17)	(vs AA/AS) 0.705
ESR1 PvuII c.453-397 T > C (rs2234693)			0.697
CC	18.8% (15)	24.1% (21)	(vs CT/TT) 0.454
CT	48.8% (39)	46.0% (40)	(vs CC/TT) 0.546
TT	32.5% (26)	29.9% (26)	(vs CC/CT) 0.740

Evaluation of whether there is an association between *FSHR* and *ESR1* SNPs and biochemical and ultrasonographic parameters in PCOS women

For PCOS patients, biochemical parameters to analyze the gonadal axes (FSH, LH, estradiol, and progesterone), total testosterone and AMH levels, and ultrasonographic data on antral follicle count were compared between different *FSHR* rs6166 and *ESR1* rs2234693 genotypes. Patients with the SS variant of the *FSHR* polymorphism had higher FSH levels on the third day of the menstrual cycle (p = 0.011). We did not find other statistically significant differences in biochemical and ultrasonographic parameters in relation to the *FSHR* or the *ESR1* genotypes. The detailed results are presented in Table 5.

**Table 5:** Results of analysis of the association between FSHR rs6166 and ESR1 rs2234693 polymorphisms and clinical and biochemical data in PCOS patients Data expressed as mean ± SD; * p < 0.05 vs control group. (1) Post-hoc: statistically significant difference between SA and SS (p-value = 0.012). PCOS: polycystic ovary syndrome; BMI: body mass index; FSH: follicle-stimulating hormone; LH: luteinizing hormone; AMH: anti-müllerian hormone; AFC: antral follicle count; MII: metaphase II; MI: metaphase I; GV: germinal vesicle.

FSHR rs6166 polymorphism								
Parameter	PCOS							
	AA		AS		SS			P-value
FSH 23^rd^ day (mUI/mL)	5.9±3.3		5.2±1.8		6.4±3.2			0.173
FSH 3^rd^ day (mUI/mL)	6.2±1.6		5.6±1.6		9.2±9.2			0.011*^(1)^
LH 23^rd^ day (mUI/mL)	12.0±6.5		12.9±10.0		14.4±18.3			0.843
LH 3^rd^ day (mUI/mL)	9.9±7.8		7.6±5.4		8.6±6.4			0.460
Estradiol 23^rd^ day (pg/mL)	96.6±68.1		119.7±96.3		103.3±50.1			0.390
Estradiol 3^rd^ day (pg/mL)	58.6±44.3		49.8±15.9		55.6±24.6			0.334
Progesterone (ng/mL)	4.1±8.9		3.6±5.4		3.7±4.3			0.710
Testosterone (ng/mL)	0.6±0.3		0.6±0.4		0.8±0.8			0.957
AMH (ng/mL)	8.3±7.2		7.6±5.4		5.2±5.7			0.459
AFC	16.9±8.1		22.7±14.0		14.4±9.9			0.357
ESR1 rs2234693 polymorphism								
Parameter		PCOS						
		CC		CT		TT	p-value	
FSH 23^rd^ day (mUI/mL)		5.7±1.8		5.4±2.8		5.8±2.0	0.524	
FSH 3^rd^ day (mUI/mL)		5.8±1.8		7.1±6.4		6.2±1.9	0.762	
LH 23^rd^ day (mUI/mL)		12.9±5.8		14.2±15.2		11.1±6.6	0.483	
LH 3^rd^ day (mUI/mL)		8.6±3.8		7.6±5.5		9.2±8.5	0.391	
Estradiol 23^rd^ day (pg/mL)		75.8±23.3		126.4±97.0		110.76.0	0.301	
Estradiol 3^rd^ day (pg/mL)		46.4±13.5		58.2±38.0		52.0±20.0	0.600	
Progesterone (ng/mL)		2.2±3.3		4.0±5.3		4.8±9.5	0.654	
Testosterone (ng/mL)		0.6±0.3		0.6±0.4		0.7±0.6	0.630	
AMH (ng/mL)		8.5±3.9		7.2±7.5		6.6±5.2	0.255	
AFC		25.2±12.7		17.9±10.9		15.9±10.9	0.080	

Evaluation of whether *FSHR* and *ESR1* SNPs influence immediate IVF outcomes in PCOS women

A comparison of an average daily dose of rhFSH, follicle count on the day of ovulation trigger, number and quality of obtained oocytes, and blastocyst number between different *FSHR* rs6166 and *ESR1* rs2234693 is presented in Table 6.

**Table 6:** Results of analysis of the association between FSHR rs6166 polymorphism and response to controlled ovarian stimulation and IVF outcomes by PCOS and control groups Data expressed as mean ± SD; * p < 0.05 vs control group; (1) post-hoc: statistically significant difference between SA and SS (p-value = 0.039); (2) post-hoc: statistically significant difference between SA and SS (p-value = 0.011). PCOS: polycystic ovary syndrome; FSHR: follicle-stimulating hormone receptor; ESR1: estrogen receptor 1; IVF: in vitro fertilization; rhFSH: recombinant human follicle-stimulating hormone; MII: metaphase II; MI: metaphase I; GV: germinal vesicle.

FSHR rs6166 polymorphism						
Parameter	PCOS					
	AA	AS		SS		P-value
rhFSH, average cumulative dose (IU)	1498.1±359.3	1425.4±474.8		1860.5±627.8		0.046*^(1)^
rhFSH, average daily dose (IU)	146.3±39.9	141.6±46.2		184.5±59.4		0.014*^(2)^
Follicle count on the day of ovulation induction						
10-15 mm	6.1±5.2	6.7±5.4		2.8±3.2		0.146
>15 mm	7.5±3.2	9.1±3.6		8.4±5.8		0.176
Total	13.6±6.6	15.7±7.5		12.8±7.8		0.285
Oocyte number						
MII	6.8±4.5	9.1±6.0		10.2±9.9		0.363
MI or GV	2.0±2.5	2.3±2.3		2.4±2.9		0.887
Degenerative or atretic oocyte	1.8±1.9	2.3±2.6		3.9±6.3		0.866
Total	10.6±7.0	13.8±7.4		16.4±15.8		0.216
Blastocyst number	1.4±2.1	1.2±1.6		2.6±3.2		0.377
ESR1 rs2234693 polymorphism						
Parameter	PCOS					
	CC		CT		TT	P-value
rhFSH, average cumulative dose (IU)	1415.0±381.8		1609.3±510.9		1470.9±529.9	0.298
rhFSH, average daily dose (IU)	139.7±41.9		158.8±51.2		146.3±49.7	0.327
Follicle count on the day of ovulation induction	1415.0±381.8		1443.7±510.9		1470.9±529.9	
10-15 mm	6.2±5.5		6.1±4.7		5.3±4.6	0.770
>15 mm	8.9±4.5		8.1±3.4		8.6±4.5	0.747
Total	15.1±7.8		14.2±6.3		13.8±8.0	0.824
Oocyte number						
MII	9.9±9.1		8.4±5.3		7.6±6.2	0.622
MI or GV	2.5±2.5		2.4±2.6		1.8±2.2	0.540
Degenerative or atretic oocyte	2.6±3.0		4.9±2.2		3.0±5.1	0.763
Total	15.0±12.6		12.8±7.7		12.5±9.9	0.687
Blastocyst number	1.7±2.3		1.4±1.9		1.6±2.4	0.981

There was a higher cumulative and daily rhFSH dose in women with the *FSHR* SS variant (p = 0.046 and 0.014, respectively). We then performed linear regression, with age, BMI, and SS vs remaining genotypes. The model was statistically significant for predicting cumulative rhFSH dose (p = 0.001), with the presence of the *FSHR* rs6166 SS variant being an independent predictor (B = 0.222, t = 2.349, p = 0.021). For *ESR1* rs2234693, the CT genotype was associated with a numerically higher rhFSH dose, but without achieving statistical significance.

No differences were observed with any polymorphisms concerning FSH dose, AFC, metaphase II oocytes, low-quality oocytes (metaphase I and atretic), and blastocyst number.

## Discussion

In this study, we evaluated women with and without PCOS. By study design, the groups with and without PCOS were homogeneous in relation to age and BMI. Nevertheless, and in agreement with what is described, these two groups had significant differences in hormonal patterns. PCOS was associated with elevated gonadotropins and testosterone, and lower estrogens and progesterone. These women also had higher antral follicle count and more oocytes obtained on average. However, there was no statistically significant difference in relation to the percentage of mature oocytes. After applying IVF techniques, the number of blastocysts obtained was similar between the two groups. These data suggest that even though PCOS women have an endocrine dysregulation that causes anovulatory infertility, a good response after COS is expected, which is in accordance with previously published data [19].

Our first question was whether *FSHR* rs6166 and *ESR1* rs2234693 were associated with PCOS risk. We did not find statistically significant differences in the distribution of each of the genotypes of *FSHR* rs6166 and *ESR1* rs2234693 polymorphisms, suggesting that in our population, neither of the studied polymorphisms is associated with PCOS risk.

Indeed, results from previous studies regarding the relationship between both *FSHR* and *ERS1* polymorphisms and PCOS present conflicting data. In a group of adolescents in Turkey, Unsal et al. did not find a different distribution of several *FSHR* polymorphisms associated with PCOS [4]. Wu et al. also failed to find an association between the *FSHR* polymorphisms and PCOS in women in the north of China, but the authors did report an association with higher levels of FSH [8]. An absence of association was also reported in Thai [20] and in Sri Lankan women [21]. More recently Wan et al. also reported an absence of association between several *FSHR* polymorphisms, including rs6166, and PCOS risk in Asian women [22]. An association between *FSHR* rs6166, but not rs6165, was reported by Gu et al. in Korean women [23]. Conversely, in an Italian cohort, Dolfin et al. did find a relationship between the rs6165 variant and PCOS risk [24]. Kim et al. found a significant association between rs6165 and rs6166 *FSHR* polymorphisms and PCOS in South Korea [11].

Regarding *ERS1*, the contribution of *ERS1* genetic variants to PCOS is also controversial. In a study from Korea, Kim et al. reported an association with the risk of PCOS [25]. Jiao et al. also found such an association in the Chinese population [26]. Conversely, Silva et al. [27], Valkenburg et al. [12], and Mir et al. [28] found no association with PCOS risk. A recent meta-analysis found no significant association between several *ERS1* polymorphisms and PCOS risk, even taking ethnicity into account [29].

It has also been hypothesized that even if there is no association between *FSHR* polymorphisms or *ERS1* and disease risk, there may be an association with its phenotype and hormone levels [12,27]. As such, we aimed to understand whether the distinctive hormonal pattern observed in these patients could be attributed to *FSHR* rs6166 or *ESR1* rs2234693 polymorphisms. The SS genotype of the *FSHR* rs6166 polymorphism was associated with higher FSH levels on the third day of the menstrual cycle. We found no other differences in evaluating the gonadal axes, AMH, or AFC between different *FSHR* rs6166 and *ESR1* rs2234693 genotypes. It has previously been reported that the SS variant of the *FSHR* rs6166 polymorphism may be less sensitive to FSH associated with higher FSH levels [30,31]. Our findings support this theory.

There has been some research on the influence of the polymorphisms evaluated in this study on IVF outcomes. As such, we also sought to understand if *FSHR* and *ESR1* SNPs influence immediate IVF outcomes in PCOS women. We did not find variations in oocyte quality or the number of blastocysts obtained in relation to these polymorphisms. We did, however, find a need for higher rhFSH dose in women with the *FSHR* rs6166 S/S variant, which may indicate a tendency toward FSH resistance in patients with these variants. Some authors have reported differences in response to COS related to *FSHR* rs6166. Jun et al. found a lower number of oocytes retrieved in association with the S/S variant [32]. Behre et al. reported lower *FSHR* sensitivity associated with this variant, but they also state that it can be overcome by increasing the rhFSH dose [33]. Loutradis et al. found an association between the AS variant and more obtained follicles and oocytes [34]. Regarding the *ERS1* rs2234693 polymorphism, there are fewer studies. However, studies with Chinese [6] and Greek women [35] suggest that the variant is associated with worse IVF results, which we did not observe in our sample.

Controversial findings in this field may be related to differences in study design and sample selection. As other authors have stated, the contribution of each gene in multifactorial diseases, such as PCOS, is small, and very large samples may be necessary to detect a small effect. Furthermore, there is the possibility of incomplete penetrance and gene-gene interactions; therefore, studies of individual genes in each population may not reveal the general pattern [31]. Nevertheless, it is possible that due to genetic heterogeneity, the same polymorphism has distinct contributions to PCOS risk and phenotype across different populations. This adds another level of complexity when defining the genetic risk factors based on a complex disease.

Our study has some limitations, mainly the relatively small sample size and lack of data on the women in whom cycle cancelation was required. The fact that the population studied is composed exclusively of women referred for fertility treatments precludes the generalization of our conclusions to all Portuguese PCOS patients. Additionally, controversial findings still exist when applying SNPs analysis in infertile populations, which may be related to the diverse populations and genotyping methods.

## Conclusions

As far as we know, this is the first study evaluating the association between *FSHR* rs6166 and *ESR1* rs2234693 polymorphisms and PCOS in the Portuguese population with infertility. Our data do not support an association between these polymorphisms and PCOS risk, phenotype, and immediate IVF outcomes. Furthermore, the *ESR1* rs2234693 polymorphism was not associated with a difference in baseline hormone values ​​or response to ovarian stimulation. Nevertheless, the SS genotype of the *FSHR* rs6166 polymorphism was associated with higher FSH levels on the third day of the menstrual cycle and also with a need for a higher cumulative dose of FSH in COS, which may reflect a lower sensitivity to FSH. Therefore, COS with higher doses of FSH may be more suitable in women with this genotype.
